# Shark: fishing relevant reads in an RNA-Seq sample

**DOI:** 10.1093/bioinformatics/btaa779

**Published:** 2020-09-14

**Authors:** Luca Denti, Yuri Pirola, Marco Previtali, Tamara Ceccato, Gianluca Della Vedova, Raffaella Rizzi, Paola Bonizzoni

**Affiliations:** Department of Informatics, Systems and Communication, University of Milano-Bicocca, Milano 20126, Italy; Department of Informatics, Systems and Communication, University of Milano-Bicocca, Milano 20126, Italy; Department of Informatics, Systems and Communication, University of Milano-Bicocca, Milano 20126, Italy; Department of Informatics, Systems and Communication, University of Milano-Bicocca, Milano 20126, Italy; Department of Informatics, Systems and Communication, University of Milano-Bicocca, Milano 20126, Italy; Department of Informatics, Systems and Communication, University of Milano-Bicocca, Milano 20126, Italy; Department of Informatics, Systems and Communication, University of Milano-Bicocca, Milano 20126, Italy

## Abstract

**Motivation:**

Recent advances in high-throughput RNA-Seq technologies allow to produce massive datasets. When a study focuses only on a handful of genes, most reads are not relevant and degrade the performance of the tools used to analyze the data. Removing irrelevant reads from the input dataset leads to improved efficiency without compromising the results of the study.

**Results:**

We introduce a novel computational problem, called gene assignment and we propose an efficient alignment-free approach to solve it. Given an RNA-Seq sample and a panel of genes, a gene assignment consists in extracting from the sample, the reads that most probably were sequenced from those genes. The problem becomes more complicated when the sample exhibits evidence of novel alternative splicing events. We implemented our approach in a tool called Shark and assessed its effectiveness in speeding up differential splicing analysis pipelines. This evaluation shows that Shark is able to significantly improve the performance of RNA-Seq analysis tools without having any impact on the final results.

**Availability and implementation:**

The tool is distributed as a stand-alone module and the software is freely available at https://github.com/AlgoLab/shark.

**Supplementary information:**

Supplementary data are available at *Bioinformatics* online.

## 1 Introduction

RNA-Seq analysis plays an important role in the biological and medical research aimed at deepening our understanding of cellular biological processes and their relationships with pathological conditions. As such, several research initiatives had the objective of producing RNA-Seq data, while a number of tools have been proposed to analyze such datasets and have gained widespread adoption in the community (Beretta *et al.*, 2017; Bray *et al.*, 2016; Denti *et al.*, 2018; Patro *et al.*, 2017; Sacomoto *et al.*, 2012; Shen *et al.*, 2014; Trincado *et al.*, 2018). Traditional approaches generally rely on RNA-Seq read mapping to the annotated gene isoforms or on the spliced alignment of reads to the genome. In the second case, spliced aligners, such as STAR (Dobin *et al.*, 2013), employ gene annotations to obtain more accurate results. While gene annotations, especially for humans, are readily available, they are not complete as they are built from healthy individuals. Thus, aberrant isoforms, which play an important role in the development of human diseases (Kahles *et al.*, 2018; Tazi *et al.*, 2009), are usually not annotated. As a consequence, *de novo* (or assembly-first) approaches (Haas *et al.*, 2013; Sacomoto *et al.*, 2012), which potentially detect novel splicing events, have been developed and have gained popularity, even for studying well-annotated organisms (Benoit-Pilven *et al.*, 2018). These approaches are more computationally demanding than traditional annotation-guided ones and pose challenging issues when facing the huge amount of RNA-Seq data that is now available. Furthermore, high-coverage samples are needed for obtaining accurate results with *de novo* approaches, especially for reconstructing low-abundance isoforms.

When the study is focused on the analysis of a pre-identified set of genes — e.g. those that are known to have a role in tumor progression — a preprocessing step that filters the input RNA-Seq reads, retaining only those likely originated by the genes of interest, could greatly reduce the size of the dataset that must be analyzed (hence speeding up the analysis) without significantly affecting the final results. Existing spliced aligners, such as STAR, could be theoretically adapted to perform the preprocessing step. However, they are aimed at obtaining accurate alignments; hence, they are not fast enough to give a significant speedup of the analysis. For example, if we trivially apply STAR using as a reference the selected gene sequences, the alignment process will take longer than using the full reference genome, since numerous attempts to align the reads sequenced from not-selected genes are performed before discarding it. Other approaches, such as some recently proposed transcript quantification tools (Bray *et al.*, 2016; Patro *et al.*, 2014, 2017) or quasi-mappers (Srivastava *et al.*, 2016), are fast enough to be adapted as preprocessing filters but they rely on isoform annotations, hence they might make errors if novel splicing events are supported by the sample, influencing any downstream analysis (Conesa *et al.*, 2016). Other *k*-mer-based approaches are promising (Almodaresi *et al.*, 2019; Sun *et al.*, 2018) but they have been designed toward different applications (e.g. indexing and searching large sequencing experiments) hence they achieve a different trade-off between computational efficiency and resulting accuracy.

For this reason, we propose an alignment-free method that solves the gene-read assignment problem without relying on existing isoform annotations: given a set of genes of interest (gene panel) and a genome-wide RNA-Seq dataset, the goal is to retain only those reads (called *relevant* reads) originating from a gene in the selected set, thus, discarding a potentially huge set of reads not relevant for the downstream analyses. We implemented the method in the tool Shark that, by relying on succinct data structures and multi-threading, is able to quickly analyze huge RNA-Seq datasets on a standard PC. We replicated two studies (Benoit-Pilven *et al.*, 2018; Trincado *et al.*, 2018) aimed at detecting differentially expressed alternative splicing events — one of the most complex and most common tasks in RNA-Seq analysis — and we found that Shark provides a preprocessing step that significantly reduces the running time and/or the memory requirements of computationally intensive downstream analyses, while not negatively impacting their results. We note that, although our method has been specifically designed for RNA-Seq data, it can also be used for genomic reads if, e.g. one is interested in finding variants located on a specific subset of genes.

## 2 Materials and methods

### 2.1 The gene assignment problem

Let Σ be a finite alphabet of size σ and let $s=c_{1},\ldots ,c_{n}$ be an ordered sequence of *n* characters drawn from Σ; we say that *s* is a string over Σ of length *n*. From a computational point of view, a *genome*, a *gene locus* and a *transcript* are strings over the alphabet {A,C,G,T}. A gene locus is a substring of the genome, whereas a transcript is a concatenation of pieces (exons) of a gene locus; an RNA-Seq sample is a set of strings (called *reads*) over the same alphabet. A RNA-Seq read is a substring of a transcript, and it is in a 1-to-1 correspondence with a gene locus, referred in the following as the *origin* of the read. Given a string *s* and a positive integer *k*, we say that a substring of *s* of length *k* is a *k*-mer. We denote by KMER(s), the multiset of all the *k*-mers of *s* (observe that the same *k*-mer might occur multiple times). As usual (Belazzougui *et al.*, 2016; Denti *et al.*, 2019; Kokot *et al.*, 2017; Marçais and Kingsford, 2011), to account for the double stranded nature of the human genome, when we refer to a *k*-mer, we implicitly refer to its *canonical form*, that is the lexicographically smaller sequence between the *k*-mer and its reverse-complement. Given a read $s=c_{1}\cdots c_{n}$, we refer to the pair $\left(c_{i},i\right)$ with the term *base*. Note that, the same character appearing at two different positions of *s* are two distinct bases. Moreover, we say that a base *b* of the read *s* is shared with the gene *g* if there exists a *k*-mer of *s* that includes *b* and is equal to a *k*-mer of *g*. We denote by SHARED(s,g), the set of the bases of *s* shared by *g*. In other words, SHARED(s,g) is the set of all the bases of *s* such that there exists a *k*-mer in the intersection between KMER(g) and KMER(s) that includes the base.

To assign a read to its origin gene, we adapt the following criterion as a proxy. A gene *g* is the putative origin of a read *s* if and only if the ratio |SHARED(s,g)|/|s| is greater or equal than a given threshold τ and for no other gene g′, |SHARED(s,g′)|>|SHARED(s,g)|. The rationale behind this criterion is to have a measure of similarity between a read and a gene without aligning them, which takes into account that introns are spliced out from transcripts and therefore from an RNA-Seq read. Observe that by this definition, and due to genome repetitions, a read may have multiple origin genes. We denote as ORIGIN(s), the set of putative origin genes of a read *s*.

We now provide a formal definition of the problem, we tackle in this article, namely the *Gene Assignment Problem*.

Problem 1 (Gene Assignment Problem). Let *S* be a set of RNA-Seq reads sequenced from a set G of genes, let $G=\{g_{1},\ldots ,g_{p}\}\subseteq G$ be a gene panel, i.e. a set of genes of interest. The *gene assignment* of *S* with respect to *G* and parameters *k* and τ is a set $A=\{S_{1},\ldots ,S_{\left|G\right|}\}$ of |G| elements such that $S_{i}\subseteq S$ is the set of reads that originate from *g_i_*, i.e. for each *s* in *S_i_* the following conditions hold: (i) $|\mathrm{SHARED}(s,g_{i})|/|s|\geq \tau$, (ii) for no other gene *g_j_*, $\left|\mathrm{SHARED}(s,g_{j})|>|\mathrm{SHARED}(s,g_{i})\right|$, and the SHARED(·,·) sets have been computed on *k*-mers.

Note that, a gene assignment is not necessarily a partition of the input sample. Indeed, a read may have more than one origin gene and some read may have no origin gene. For ease of presentation, in the rest of the article, we will refer to this problem as the gene assignment of *S* with respect to *G*, without specifying *k* and τ.

Note also that the Gene Assignment Problem here defined is robust to sequence polymorphisms. Indeed, from the viewpoint of the definition of the problem, sequence polymorphisms are indistinguishable from sequencing errors, and parameters *k* and τ allow (as we show in Section 3) to tweak the accuracy toward better specificity (allowing less errors) or better sensitivity (allowing more errors).

The algorithmic approach, we propose to solve this problem uses two well-known data structures that we will now briefly introduce. A *bit vector* is a sequence of binary values that supports rank and select queries in constant time using additional sublinear space. Let *B* be a bit vector and let *i* be a positive integer, $\mathrm{ran}k_{d}(B,i)$ is the number of values equal to *d* in the portion B[1,i) of *B*, whereas $\mathrm{selec}t_{d}(B,i)$ is the position of the *i*-th value of *B* set to *d*. Clearly, $\mathrm{ran}k_{d}(B,i)$ is not defined if *i* is greater than the size of *B* and $\mathrm{selec}t_{d}(B,i)$ is not defined if fewer than *i* values of *B* are set to *d*. A *Bloom filter* (Bloom, 1970) is a probabilistic data structure used to answer membership queries. It consists of a bit vector of fixed size *m* and *z* hash functions, each one mapping an object to a position of the bit vector. Adding an object to a Bloom filter consists in setting to 1 the bits at the positions of the bit vector computed by the hash functions for that object. Testing if an object is in the set consists in checking whether all the bits at the positions computed by the hash functions for that object are set to 1. Due to hash collisions, an element may be reported as present even though it is not in the set, resulting in a false positive. Anyway, a low false positive rate can be achieved via a suitable choice of the bit vector size and the number of hash functions.

### 2.2 Algorithm

In this section, we describe the algorithmic approach, we propose to solve the computational problem introduced in the previous section. The algorithm for computing the gene assignment of an RNA-Seq sample *S* with respect to a set *G* of genes is composed of two steps: first, for each read *s* in the sample, we compute the set ORIGIN(s) of its origin genes, then, we derive the gene assignment of *S* from those sets by grouping together reads with the same origin gene.

An efficient solution to this problem essentially requires that we index the gene sequences. A simple procedure stores a dictionary that maps each *k*-mer appearing in at least one gene to the genes in which it occur, and then use it to map the *k*-mers of the reads to the genes, determining the origin genes read by read. Although effective, this approach would require an excessive amount of memory to store the dictionary if we need to track a significant amount of *k*-mers of the genes, especially since we have to store explicitly the *k*-mers sequences. For this reason, we designed a novel data structure that couples efficient access with small space usage, albeit introducing some false positives.

The data structure we propose to efficiently compute a gene assignment consists of a Bloom filter *BF*, a bit vector *P* and a vector of integers *I*. We use the three components of the data structure as follows: the Bloom filter stores the set of *k*-mers of the genes in *G*, the vector of integers compactly stores the subset of genes in which each *k*-mer appears and the bit vector tags the boundaries of the different subsets in the integer vector.

To build this data structure, we designed a three-step process, also presented in Algorithm 1. First, we associate to each gene in *G* an incremental ID and store the gene-ID mapping in a dictionary *GENEMAP*, which is then given as argument to Algorithm 1. Notice that, we achieve an important reduction in memory usage by storing only the gene-ID instead of its entire sequence.

Then, we scan the gene sequences and we store each *k*-mer in *BF* by using a single hash function *H*, mapping each *k*-mer to a specific position in the Bloom filter *BF* (lines 1–5). Once we complete this scan, *BF* stores the set of *k*-mers of all input genes. Since the number of *k*-mers indexed in the Bloom filter is significantly smaller than the size of the Bloom filter, using a single hash function improves efficiency and only slightly degrades accuracy, as shown by various works (Denti *et al.*, 2019; Sun *et al.*, 2018). Efficiency improves in this setting because using multiple hash functions increases the number of random memory accesses, thus increasing the amount of cache misses.

For each 1 in *BF*, we create an empty list *L_r_* (lines 6–7), where *L_r_* corresponds to the *r*-th bit stored in the Bloom filter. We will then use *L_r_* to store a set of back-references to the genes where each associated *k*-mer appears. At the end of this step, each 1 in *BF* is associated with a subset of genes back-references (represented as a list of IDs) stored in memory. Then, we scan all the *k*-mers in each gene of *G*, we compute the corresponding 1 in *BF* and (via a rank) the corresponding list *L_r_* to which the gene-ID must be appended (lines 8–12). Finally all duplicates are removed from the lists *L_r_*.

The third step consists of concatenating the lists *L_r_* to obtain the integer vector *I* that contains all back-references. At the same time (lines 15–19), we build the boundary vector *P*, which has a 1 in each position $\sum_{i=1}^{r}|L_{i}|$ where r≤ℓ (i.e. the 1s mark the end of each list).

**Fig. 1.**
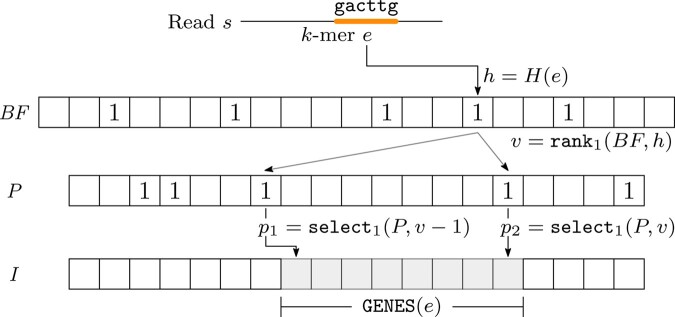
Relation between the Bloom filter *BF*, the bit vector *P*, and the vector *I*. To retrieve the identifiers of the genes containing a *k*-mer *e* (gacttg in the figure), we compute its image *h* through *H* and, if BF[h] is the *v*-th 1 of *BF*, the positions of the (v−1)-th and the *v*-th 1 of *P*, denoted as *p*_1_ and *p*_2_, respectively, can be found via rank and select operations. The interval of *I* from $p_{1}+1$ to *p*_2_ stores the set GENES(e) of the indices of the genes containing the *k*-mer *e*

The example in Figure 1 describes how this data structure is queried to retrieve the identifiers of the genes associated to a given *k*-mer. First, the hash function *H* maps the 5-mer *gactgg* to position *h*. Since a 1 is at position *h* of *BF*, we suppose that the *k*-mer is in it and we compute how many 1s appear before *h* computing in constant time $\mathrm{ran}k_{1}(BF,h)$. Then, since the 1 stored in *h* is the *v*-th 1 of *BF*, i.e. the *k*-mer is the *v*-th element according to the order of *k*-mers given by *H*, we retrieve the positions of the (v−1)-th and the *v*-th 1 in *P* using $\mathrm{selec}t_{1}$. Those positions are the boundaries on *I* of the subset of genes mapped to the *k*-mer.

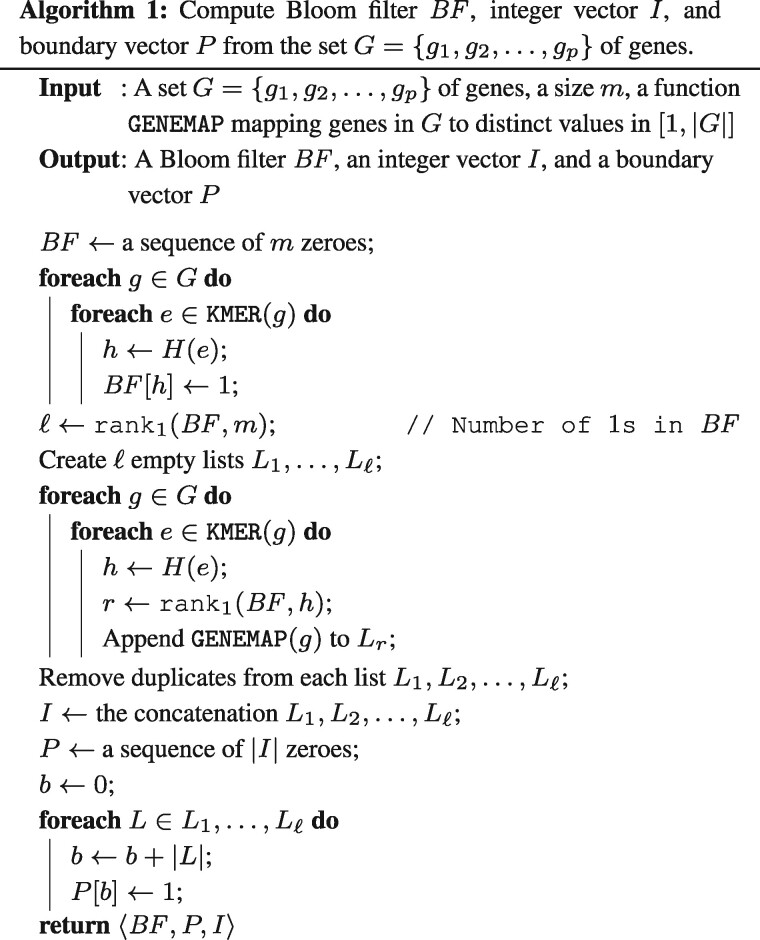

Once we have built the data structure D(G)=〈BF,P,I〉, as described above, we iterate over each read of the sample and we query *D*(*G*) to compute the set of its origin genes. For each read *s*, the procedure scans the multiset KMER(s) of *k*-mers of *s* from left to right and maintains, for each gene *g*, the rightmost position $pos_{g}$ of a base of *s* shared by *g* (or −1 if such a base does not exist) and the variable $\mathrm{car}d_{g}$ that contains the number of bases of *g* that are shared with some *k*-mers of *s* seen so far — at the end, $\mathrm{car}d_{g}$ will be equal to |SHARED(s,g)|. Let *e* be the *i*-th leftmost *k*-mer of *s* and let GENES(e) be the set of genes containing *e* [obtained by querying *D*(*G*) as illustrated in Fig. 1]. Then, for each gene *g* in GENES(e), the procedure updates $\mathrm{car}d_{g}$ by adding the number of new bases covered by *e* and shared between *s* and *g* (computed as $\min\{k,i+k-pos_{g}\}$), and sets $pos_{g}$ to the last position in *s* that is in *e*, equal to *i* +* k*. At the end of the scan, each $\mathrm{car}d_{g}$ is clearly the cardinality of SHARED(s,g) and computing the origin gene of the read is trivial.

### 2.3 Computational complexity

Let us assume that *H* is computed in constant time since *k* is fixed and, for practical purposes, it is not >32 (hence each *k*-mer can be represented in a single memory word). Constructing the index data structure D(G)=〈BF,P,I〉 requires $O(|BF|+\sum_{g\in G}|g|)$ time (assuming that $\mathrm{ran}k_{1}$ is computed in constant time) since duplicates (if any) in each list *L_i_* are adjacent. Querying *D*(*G*) for the set of origin genes of a single *k*-mer *e* requires O(|GENES(e)|) (assuming that also $\mathrm{selec}t_{1}$ is computed in constant time). Computing the origin gene of a read *s* requires $O(\sum_{e\in \mathrm{KMER}(s)}|G\mathrm{ENES}(e)|)$ that is trivially upper bounded by O(|s|·|G|).

### 2.4 Implementation

The method described in Section 2.2 has been implemented in C++ and is freely available at https://github.com/AlgoLab/shark and published on BioConda (Grüning *et al.*, 2018). The program uses the implementation of bit vectors and of the associated rank and select operations provided by sdsl (Gog *et al.*, 2014).

The tool, called Shark, takes as input a FASTA file containing the set of gene regions of interest and an RNA-Seq sample in FASTQ format. For each read of the sample, the tool computes its set of (putative) origin genes (computing the gene assignment is then trivial). It is possible to tune the computation of the gene assignment by setting the following parameters: the *k*-mer size *k*, the confidence τ and the size *m* of the Bloom filter. The tool also allows to discard *k*-mers spanning bases whose quality is less than a given threshold *q*.

## 3 Results

To assess our method, we performed two different experimental analyses on simulated and real data. A first exploratory analysis on simulated data was performed to test the accuracy and the efficiency of Shark, especially with respect to its input parameters *k*, τ and *q*. Furthermore, we also investigated the influence of read length and gene size on the accuracy of Shark. The goal of the second part of the analysis was to evaluate, on real data, the effectiveness of Shark in speeding up four different pipelines (three mapping-first pipelines, Section 3.2, and an assembly-first pipeline, Section 3.3) for a common task in RNA-Seq data analysis, namely differential analysis of alternative splicing events, without affecting the results on the selected genes. All the experiments were performed on a 64 bit Linux (Kernel 4.4.0) system equipped with four 8-core Intel^®^ Xeon 2.30 GHz processors and 256 GB of RAM. Information on how to reproduce the experiments are available at https://github.com/AlgoLab/shark_experiments in the form of a Conda environment and several Snakemake workflows (Köster and Rahmann, 2012). To reproduce the assembly-first experiments, we refer the reader to http://kissplice.prabi.fr/pipeline_ks_farline/.

### 3.1 Simulated data

We performed an exploratory analysis on simulated data to test the accuracy and the efficiency of our method, especially with respect to its input parameters *k*, τ and *q*. To this aim, we considered the 9403 genes of Human chromosomes 1, 17 and 21 (*Ensembl release 97*) (Cunningham *et al.*, 2019) and we simulated an RNA-Seq sample of 10 million 100 bp-long single-end reads using Flux Simulator (Griebel *et al.*, 2012) (see Supplementary Fig. S2 for the simulation parameters). From the full set of genes, we selected 10 random subsets of 100 genes, producing 10 different instances. For each instance, Shark indexed the gene panel, consisting of the 100 considered genes, and then filtered the entire simulated RNA-Seq sample with respect to it. To assess accuracy and efficiency of Shark with respect to input parameters, we ran Shark with any combination of k∈{13,17,23,27,31}, τ∈{0.2,0.4,0.6,0.8} and q∈{0,10,20}. Furthermore, we tested Shark also by dropping reads that are assigned to more than one origin gene (‘single mode’). In such a mode, Shark completely discards ambiguous assignments (if a read can be assigned to two or more genes, then no association at all is given) and it is useful in these experiments to better understand the accuracy of Shark. The size of the Bloom filter was set to 1 GB (preliminary experiments showed that larger Bloom filters did not improve the accuracy of the prediction). Shark was allowed to use four threads to speed up the computation.

The accuracy of Shark in computing the gene assignment was measured in terms of *precision* and *recall* as follows. Since the input reads have been simulated, we know the actual origin gene of each read. Let G⊆G be the subset of genes of interest and G be the set of genes which the set of reads *S* have been simulated from. Let $A=\{S_{1},\ldots ,S_{\left|G\right|}\}$ be the output of Shark on *S* and *G* (we recall that $S_{i}\subseteq S$ is the subset of reads assigned to *G_i_* by Shark). Then, each $s\in S_{i}$ is a *true positive* (tp) if *s* was simulated from *g_i_*, and it is a *false positive* (fp) otherwise. Finally, each read s′∈S simulated from gene *g_i_* such that s′∈Si is a *false negative* (fn). More intuitively, we consider each read associated to the correct origin gene of interest as a true positive and each read assigned to a gene of interest that was not simulated from that gene as a false positive. We note that with this definition, false positives occur in two slightly different cases: (i) when a read is simulated from a gene of interests and it is assigned to a different gene of the panel and (ii) when a read is simulated from a gene not in the gene panel and it is assigned to any gene in the gene panel. Finally, we consider as false negative each read simulated from a gene in the gene panel that was not assigned to the correct gene. Notice that, when Shark is run in multiple mode a read might be assigned to more than one gene, thus a read with two assignments (one to the correct gene and one to another gene) might induce both a true positive and a false positive. Moreover, when a read is assigned only to a wrong gene, it induces both a false positive (because it was assigned to the wrong gene) and a false negative (because it was not assigned to the right gene). Then, precision is defined as $P=\frac{tp}{tp+fp}$, while recall as $R=\frac{tp}{tp+fn}$. Efficiency was measured in terms of running time and memory peak (using the/usr/bin/time system tool).

**Fig. 2.**
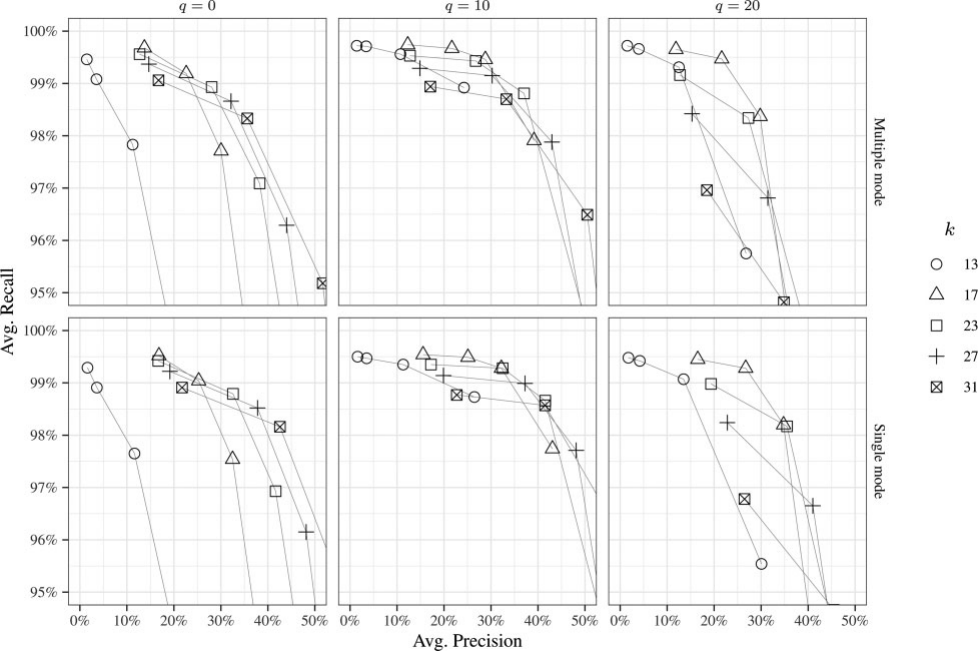
Accuracy results — exploratory analysis. Accuracy is shown in terms of average precision and average recall obtained across the 10 performed runs. Lines connect data points with τ=〈0.2,0.4,0.6,0.8〉

Figure 2 reports the precision/recall curves for the different combinations of parameters (see Supplementary Table S1 for the entire set of results). The first important observation is that Shark achieves a good accuracy in terms of recall. Indeed, for sensible choices of the parameters, the recall is at least 99%, i.e. on average at most 1% of the reads that originated from the chosen subset of genes was discarded. The second observation is that the choice of the values for the parameters *k* and τ allows to achieve different trade-offs between precision and recall. Indeed, as *k* (or τ) increases, Shark becomes more precise (at the expense of recall).

The third observation is that Shark (as expected, since it is a *k*-mer-based approach) is sensitive to sequencing errors. Indeed, if low-quality bases are not filtered out (*q* = 0), the recall rapidly decreases under 98% as τ increases. However, the simple approach of filtering out low-quality bases allows to reduce the loss of recall as the precision sensibly increases. For example, for *q* = 10 (i.e. bases with quality <10 are not considered) and *k* = 17, when τ increases from 0.4 to 0.6, we have that the precision gains 7.13 percentage points (from 21.67% to 28.80%) while the recall only decreases of 0.21% points (from 99.67% to 99.46%). Interestingly, an aggressive low-quality filter (*q* = 20) generally decreases the accuracy of Shark. This is probably due to the fact that, under this setting, the set of *k*-mers extracted from each read is too small for reliably finding its origin gene.

As explained in Section 2.2, more than one gene can be assigned to a single read. To assess how these ambiguous assignments influence the accuracy, we ran Shark excluding any multiple assignment (‘single mode’). The accuracy of Shark in ‘single mode’ compared with the original ‘multiple mode’ is sensibly higher in terms of precision and slightly lower in terms of recall. For example, for *k* = 17, τ=0.6 and *q* = 10, in multiple mode the precision is 28.8% while in single mode it is 32.2%, whereas the recall is 99.46% and 99.28% in multiple and single mode, respectively. Also in this case, keeping or discarding ambiguous assignments is a user’s choice, depending on the choice between higher recall or higher precision.

This experiment shows how accuracy is determined by the choice of the parameters. The results suggest that a good trade-off between precision and recall can be achieved with *k* = 17, τ=0.6 and *q* = 10.

In terms of computational requirements, Shark never required more than 1.5 GB of RAM (Supplementary Table S1), that is an amount of memory nowadays available on any desktop or laptop computer. Furthermore, using four threads, it never required more than 1 min to complete and, in particular, it never required more than 40 s for k≥17 (Supplementary Fig. S1). Albeit this experiment has been performed on a server platform, we expect that the running time will be practically negligible even on standard computers.

We also investigated the influence of read length and gene size on the accuracy and on the computational requirements of Shark (Supplementary Section S2). We observed that neither the read length nor the gene size affects negatively the overall accuracy of Shark. Furthermore, Shark never required more than 2 min and 5 GB of RAM to complete any analysis.

We also investigated how the number of genes of interest, i.e. the size of the input gene panel, affects the accuracy and efficiency of Shark. To this aim, we considered the 9403 genes from the 3 chromosomes analyzed in the previous experiments and we created 7 different gene panels of increasing sizes: from 100 genes to 9403 genes. We note that considering the largest gene panel is equivalent to filtering the reads with respect to the entire set of genes they were sequenced from (indeed, in that case, G=G).

We filtered with Shark, the 10 M single-end read sample simulated with Flux Simulator with respect to each panel and we evaluated the accuracy and efficiency of Shark as done in our previous analysis. We ran Shark with four threads in both single and multiple modes, setting *k* = 17, τ=0.6, *q* = 10, and the size of the Bloom filter to 1 GB. Table 1 reports the results of this analysis. First of all, we observe that, by increasing the input panel size, Shark behaves differently if ran in single or multiple mode. Indeed, increasing the number of genes of interest allows Shark (ran in multiple mode) to increase its precision as well (from 20% to 69%), while not affecting its recall, which remains really high (∼99%). The increase in precision can be explained by the fact that, as the gene panel becomes larger, Shark is able to associate each read to the correct gene, while, if the correct origin gene is not in the panel, Shark associates the read to a different (but similar) gene of the panel. This hypothesis is further confirmed by the data on single mode. Indeed, as the gene panel size increases, Shark (single mode) is able to achieve a high level of precision (∼99% with the largest gene panel tested) at the expense of a lower recall that decreases from 99% to 75%. This was expected since the gene panel also contains similar genes (e.g. overlapping genes, albeit on opposite strands), thus associations to those genes become ambiguous and are discarded in single mode. A further analysis of accuracy with respect to the similarity among the genes of interest is presented in the accompanying repository (https://github.com/AlgoLab/shark_experiments) but proved to be elusive due to the interplay of relative expression of overlapping genes (among the other factors).

**Table 1.** Accuracy and efficiency results — varying gene panel sizes

	Shark (multiple mode)				Shark (single mode)				RapMap				Puffaligner			
Gene panel size	*P*	*R*	Time (s)	RAM (GB)	*P*	*R*	Time (s)	RAM (GB)	*P*	*R*	Time (s)	RAM (GB)	*P*	*R*	Time (s)	RAM (GB)
100	19.6	99.7	27	1.41	21.9	99.7	33	1.41	21.5	99.1	51	0.20	3.4	99.3	47	0.22
250	45.7	99.7	35	1.43	50.3	99.2	36	1.43	52.1	99.0	63	0.28	6.6	99.3	63	0.27
500	54.2	99.7	58	1.46	60.4	98.1	58	1.46	60.8	99.1	79	0.48	7.1	99.4	90	0.36
1000	58.6	99.6	103	1.57	66.6	98.4	96	1.57	65.6	98.9	110	0.87	9.1	99.3	120	0.43
2500	60.7	99.7	241	1.86	73.7	91.7	248	1.86	66.9	98.9	174	1.81	13.6	99.3	184	0.70
5000	65.2	99.6	441	2.47	84.7	85.0	492	2.47	71.9	98.8	325	3.79	26.2	99.3	277	1.00
10 000	68.9	99.6	898	3.38	99.9	75.1	934	3.38	75.5	98.7	526	6.37	64.7	99.3	431	1.55

*Note*: Accuracy is shown in terms of precision (*P*) and recall (*R*), while efficiency in terms of running time (Time, in seconds) and peak memory usage (RAM, in GB).

The experiment confirmed that Shark is efficient: on the largest gene panel, it required ∼15 min and 3 GB of RAM, an amount nowadays available on any PC.

To better evaluate Shark accuracy and efficiency, we compared it against RapMap (Srivastava *et al.*, 2016), a quasi-mapper, and Puffaligner, an aligner based on compacted colored de Bruijn graphs (Almodaresi *et al.*, 2018). Although these tools are not designed to directly compute a gene assignment and the corresponding partitioning of the input RNA-Seq sample, they can be used to perform very fast read alignment that can be post-processed to extract the corresponding gene assignment. We provided the gene sequences to RapMap whereas we provided both the gene sequences and the gene transcripts to Puffaligner (as suggested in the project documentation). We ran both the tools with four threads. To compute their precision and recall, we adopted the same measures used to evaluate Shark.

Table 1 reports the results of this comparison. First of all, we observe that RapMap and Puffaligner behave more similarly to Shark ran in multiple mode than to Shark ran in single mode. Indeed, both the aligners report primary and secondary alignments and in our analysis, we considered both of them (but only an alignment per gene). Instead, if we consider only the primary alignments (data not shown), we observe a straight drop in the recall of the two tools: most of the correct assignments are indeed derived from secondary alignments. Furthermore, since RapMap and Puffaligner are not able to compute spliced alignments, we relaxed any additional check performed by these tools in order to accept even low-quality alignments (please refer to https://github.com/AlgoLab/shark_experiments for the list of parameters that have been used). Otherwise, we would have observed lower levels of recall.

In this non-standard setting, we were able to let RapMap and Puffaligner achieve a very high recall (∼99%). Anyway, Shark is able to achieve even better recall than the other two tools for all panel sizes.

Surprisingly, precision of Puffaligner is significantly lower than that of Shark and RapMap (∼6 times lower) for all panel sizes but the largest one. In our opinion, this result suggests that Puffaligner, in its current form and despite its merits as aligner, is not a suitable choice for computing a gene assignment.

RapMap is slightly more precise than Shark for all panel sizes but the smallest one, at the expense of a slightly less recall. We remark that, for the specific purpose Shark has been designed, achieving better recall is more important than achieving better precision, since discarding potentially relevant reads may introduce biases in the downstream analysis whereas lower precision will only lead to lower improvements in the running times.

In terms of memory, all the tools were able to complete the analysis using <4 GB of RAM. The only exception is RapMap on the largest gene panel, which required more than 6 GB of memory (while Shark and Puffaligner used 3.4 and 1.6 GB, respectively). On gene panels composed by at most 1000 genes, Shark, which has been designed for panels whose size is in this range, is slightly faster than the other two tools.

We remark that RapMap and Puffaligner were run with non-standard settings in order to achieve levels of recall comparable to those of Shark. However, especially for RapMap, this means that alignments of only a rather small portion of the read were accepted. Otherwise, since neither RapMap nor Puffaligner computes spliced alignments, they would not accept reads mapping to a splice junction. For this reason, we argue that, as the read length increases, Shark would be able to assign reads more accurately than the other twos. Indeed, Shark would map all the *k*-mers to the gene sequences, whereas RapMap (and Puffaligner if novel exons are present) would keep only a portion of the read falling inside an exon.

These results are promising and we believe that Shark can be effectively used to filter an RNA-Seq sample with respect to large gene panels. However, we stress that the main goal of Shark is to filter a set of reads with respect to a limited number of genes of interest in order to speed up their downstream analysis. On this kind of instances, Shark is slightly faster and has consistently higher recall than other approaches. Running Shark on a large gene panel (or even on the entire set of annotated genes), though feasible, may not significantly reduce the size of the input sample, thus not leading to any speedup in the following RNA-Seq analysis, especially if the analysis is based on fast and efficient pipelines.

### 3.2 Replication of a mapping-first differential AS analysis

In the second part of our experimental evaluation, we partially replicate the analysis of a real RNA-Seq dataset performed in Trincado *et al.* (2018) in order to assess the effectiveness of our tool in speeding up state-of-the-art pipelines for differential analysis of alternative splicing.

We considered the three pipelines based on SplAdder (Kahles *et al.*, 2016), rMATS (Shen *et al.*, 2014) and SUPPA2 (Trincado *et al.*, 2018). The first two tools analyze the RNA-Seq alignments computed by a spliced aligner, while the last one — SUPPA2 — analyzes the transcript quantifications computed by Salmon. In the following, we will refer to the three pipelines only by the name of the tools for the differential analysis, i.e. SplAdder, rMATS and SUPPA2. We remark that the aim of this part is not to evaluate the accuracy of the results of these pipelines, but to verify (i) whether their findings are affected by the preprocessing step performed by Shark and (ii) how much Shark can speed up their analyses. As mentioned in Section 1, pipelines based on STAR cannot be trivially speeded up by restricting the reference to the selected genes, while others — such as SUPPA2 — likely run faster if the reference is restricted. However, since SUPPA2 already uses a modest amount of computing resources, the benefits of restricting the reference (or of using Shark, as we will show) will be less dramatic.

The dataset used in this evaluation consists of a set of six paired-end RNA-Seq samples (GEO accession number *GSE59335*): three samples obtained before and three obtained after the double knockdown of two splicing regulatory proteins, namely TRA2A and TRA2B (Best *et al.*, 2014). Each sample contains between 22 and 25 million paired-end reads of length 101 bp. We decided to test the tools on this dataset since in the study 83 exon skipping events have been validated experimentally by RT-PCR, thus we can use such events as a ground truth to assess the effect of using Shark as preprocessing step.

In this analysis, we ran Shark setting *k*=17, τ=0.6, *q*=10 and the Bloom filter size to 1GB. We chose these values since, as proved in the previous analysis, they achieve a good trade-off between precision and recall. All tools were ran with their default parameters (allowing up to four threads), while the spliced alignments required by the first two pipelines were computed by STAR (Dobin *et al.*, 2013) in two-pass mode.

We initially computed the differentially spliced events with the three aforementioned pipelines considering the original RNA-Seq samples and then we repeated the analysis considering the RNA-Seq samples preprocessed with Shark on the 82 different gene regions involved in the 83 RT-PCR validated events. On average, the filtered samples contain ∼2.3% of the original reads. Supplementary Table S3 reports the differences in terms of number of reads and uncompressed file size between the original samples and the samples filtered by Shark.

We considered the 83 alternative splicing events validated by RT-PCR and we evaluated if the ability of the three pipelines in detecting such events is affected by the preprocessing step performed by Shark. Table 2 reports the results of this analysis.

**Table 2.** Accuracy and efficiency of the three pipelines for differential analysis of alternative splicing on the original samples compared with those obtained on the samples filtered by Shark

	RT-PCR events		Time	RAM
Pipeline	All	P-value<0.05	(min)	(GB)
rMATS	78	63	328	33.9
Shark + rMATS	78	63	154	33.9
SplAdder	56	—	915	33.9
Shark + SplAdder	56	—	351	33.9
SUPPA2	66	44	117	1.7
Shark + SUPPA2	66	51	42	1.7

*Note*: Accuracy is evaluated in terms of the number of RT-PCR validated events detected by each pipeline (over a total of 83 RT-PCR validated events). Efficiency is evaluated in terms of running time and maximum memory usage.

The first observation is that all the pipelines detected the same RT-PCR validated events in both the considered scenarios (i.e. on the original samples and on the filtered ones), confirming that the preprocessing step performed with Shark does not affect the accuracy of their differential splicing analysis. More precisely, out of the 83 RT-PCR validated events, rMATS detected 78 differentially spliced events, SUPPA2 detected 66 events, and SplAdder detected 56 events, under both conditions.

If we restrict our analysis only to events reported as statistically significant by each tool (i.e. the events with *P*-value smaller than 0.05), the results follow the same trend, further confirming that the preprocessing step does not negatively impact the differential analysis. Indeed, rMATS reported the highest number of events (63) followed by SUPPA2. Interestingly, SUPPA2 identified seven additional significant events when considering the samples preprocessed by Shark w.r.t. the 44 events detected on the original samples. A manual inspection of the events in the two scenarios revealed that the differences between the respective *P*-values were rather small. On the other hand, in both scenarios SplAdder reported all the events with a *P*-value close to 1 (hence not significant).

We also investigated the effect of filtering on the intermediate results of the pipelines. As an example, Supplementary Figure S3 compares the outputs obtained by the transcript quantifier Salmon (one of the steps of the SUPPA2 pipeline) on the full and on the filtered dataset. Albeit the absolute values of the quantification differ in magnitude, the Pearson’s correlation coefficient between all the three pairs of series is high (≥0.998), confirming, as expected, that the outcome of any differential analysis performed on the filtered dataset should not be affected by the filtering process.

The second important observation is that, as expected, preprocessing the input samples with Shark makes the three pipelines faster. Indeed, Shark (which required <5 min to process each sample) allows all those pipelines to complete their analysis in around half the time. More precisely, rMATS took 2.5 h (saving 3 h), SplAdder completed its analysis in <6 h (saving 9.5 h) and SUPPA2 took only 40 min instead of 2 h. The difference of the running times in the two scenarios is important (especially for rMATS and SplAdder), even on the relatively small dataset, we considered here. Notice that, the running time of Shark is linear in the number of input reads, and we only read once the set of reads. This fact implies that larger datasets (e.g. with more replicates or across several conditions or with higher-coverage samples) should show an even larger (absolute) reduction of running times of the complete analyses. While STAR and Salmon perform an indexing procedure only once, Shark indexes the input gene regions for each sample — hence, a more refined implementation that builds the index of the gene sequences only once could save even more time, especially for a larger number of samples.

Lastly, this experiment shows that peak memory usage is almost unaffected by Shark. Indeed Shark required <1.5 GB of RAM to process the input samples, which is significantly less than the peak memory usage of rMATS and SplAdder (33.9 GB) and comparable to that of SUPPA2 (1.7 GB). In particular, the peak memory usage for rMATS and SplAdder is reached in the alignment step. In this step, STAR loads the entire genome index in memory, hence its memory usage is largely independent on the input sample size.

We then investigated how to reduce the memory usage of STAR, that is the most memory intensive step of the pipelines we tested, to allow any pipeline to be run on standard machines typically equipped with <32 GB of RAM. When low memory usage is required, it is possible to reduce the memory usage of STAR by using its option for constructing a sparse index, namely --genomeSAsparseD, at the expense of increasing its running times (according to the tool documentation). However, since, we showed that Shark greatly reduces the input sample size and hence the time required by the alignment step, we expect that the resulting pipeline should be still faster than the classic one on the original samples. To evaluate our claim, we ran the two STAR-based pipelines multiple times on both the original samples and the samples preprocessed with Shark, each time increasing the sparsity of the STAR index. Table 3 reports the results obtained with sparsity equal to 8—the lowest value that allows to keep memory usage under 16 GB, an amount nowadays common on standard PCs.

**Table 3.** Accuracy and efficiency of the STAR-based pipelines for differential analysis of alternative splicing on the original samples compared with those obtained on the samples filtered by Shark

	RT-PCR events		Time	RAM
Pipeline	All	P-value<0.05	(min)	(GB)
rMATS	78	63	632	15.7
Shark + rMATS	78	63	138	15.7
SplAdder	56	—	1220	15.7
Shark + SplAdder	56	—	326	15.7

*Note*: The results have been obtained with --genomeSAsparseD=8 — a parameter that affects the sparsity of the index built and used by STAR. Accuracy is evaluated in terms of the number of RT-PCR validated events detected by each pipeline (over a total of 83 RT-PCR validated events). Efficiency is evaluated in terms of running time and maximum memory usage.

The first observation is that the sparsity of the index built by STAR does not affect the accuracy of the downstream pipelines for the differential analysis of alternative splicing. Indeed, both rMATS and SplAdder detected the same RT-PCR validated events in all the runs we performed.

The second observation is that increasing the sparsity of the index allows to reduce the memory usage of each pipeline, from more than 33 GB to <16 GB. Moreover, as expected, the sparsity of the index greatly affects the overall running times of the pipelines when they consider the original RNA-Seq dataset (+93% for rMATS and +33% for SplAdder, see Table 3 compared with Table 2). On the other hand, since the filtered dataset is considerably smaller than the original one, the sparsity of the index does not sensibly affect the running times of the STAR alignment step and, hence, of the pipelines when run on the filtered dataset.

### 3.3 Replication of an assembly-first differential as analysis

In the third part of our experimental evaluation, we focused on the replication of an assembly-first differential alternative splicing analysis. Assembly-first approaches are based on first assembling the RNA-Seq reads depending on their overlaps and then aligning the assembled sequences to the reference genome. Benoit-Pilven *et al.* (2018) show that mapping-first approaches (as those considered in the previous section) and assembly-first approaches (as the one considered in this section) are complementary, in the sense that, while they agree on the vast majority of their findings, each one is able to detect specific cases that the other one is not able to report (lowly-expressed variants for mapping-first approaches and novel variants for assembly-first approaches). We want to point out that assembly-first approaches are, in general, computationally demanding, thus filtering reads using Shark can be highly beneficial in order to speed up such an analysis and reducing the peak memory usage. Furthermore, we highlight that our filtering approach does not perform alignments (or pseudo-alignments) to the transcript sequences. As a consequence, Shark should not filter out reads supporting novel variants, which are mainly detected by assembly-first approaches. Hence, it is reasonable to use it as a preprocessing step of an assembly-first pipeline.

To this aim, we partially replicated the analysis performed by Benoit-Pilven *et al.* (2018) using the assembly-first KisSplice pipeline on an RNA-Seq dataset of the MCF-7 breast cancer cell line (GEO accession number *GSE94372*). The dataset is composed of two biological replicates on two conditions: a control condition and the depletion of two RNA helicases (DDX5 and DDX17). Each sample contains between 32 and 35 million paired-end reads of length 125 bp.

Similarly as we did in the previous section, we focused on the subset of 48 genes for which the authors performed an experimental RT-PCR validation of a differential alternative splicing event detected by at least one pipeline of the twos they used in the work.

As performed in the analysis we replicated, raw reads were preprocessed by trimming and removing the adapters according to standard quality control filters. The resulting dataset was then analyzed using the commands reported at http://kissplice.prabi.fr/pipeline_ks_farline/ on the full dataset and on the dataset filtered with Shark. Shark was ran setting *k* =17, τ=0.6, *q*=10 and the Bloom filter size to 1 GB.

We considered all the differentially spliced events occurring in the 48 genes for which an experimental validation has been performed. Notice that, differently from the previous section, we did not consider only the RT-PCR validated events since they were not unambiguously reported, hence it was not possible to focus only on them.

**Fig. 3.**
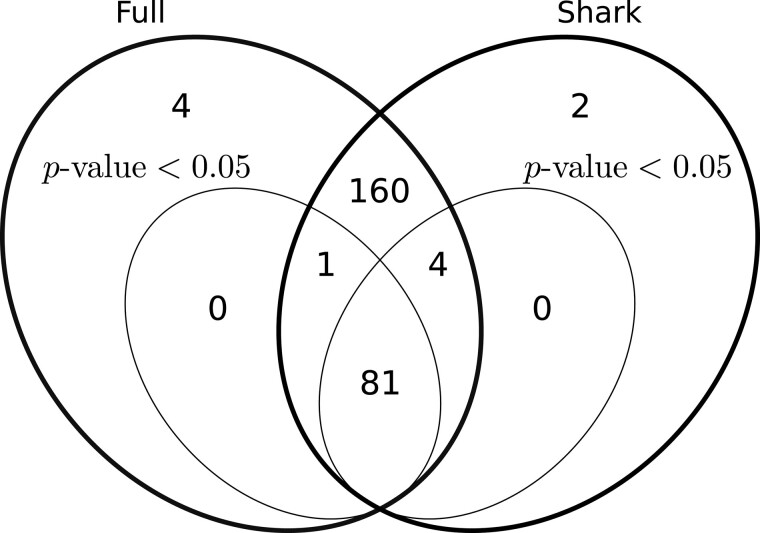
Comparison of differential alternative splicing events mapping to one of the 48 selected genes as predicted by KisSplice on the full dataset (left oval) and on the dataset filtered by Shark (right oval). Inner ovals represent the events predicted with P-value<0.05

Figure 3 reports the comparison between the events reported on the full dataset (and then keeping only those mapping to one of the 48 selected genes) and those reported on the filtered dataset. The first important observation is that the predicted events almost perfectly coincide. Indeed, 246 (97.6%) events were reported on both the full dataset and on the filtered dataset. Only four (1.6%) events were predicted exclusively on the full dataset while two (0.8%) events were predicted exclusively on the filtered dataset. If we focus on the events reported as statistically significant (P-value<0.05), 81 of them were reported on both dataset, a single event was reported as statistically significant on the full dataset but as not statistically significant on the filtered dataset, while 4 were reported as statistically significant on the filtered dataset but as not statistically significant on the full dataset. Manual inspection of the differences revealed that the reported Percent Spliced In ( ΔPSI) for these events are highly similar. Interestingly, no event reported as statistically significant on a dataset was missing in the other dataset (albeit it could be reported as not statistically significant).

In terms of running times, Shark allows to achieve a considerable speed up (Supplementary Table S4). Indeed, the total running time on the full dataset (after trimming) is 26 h and 37 min, while on the filtered dataset the pipeline (including the filtering step) took 3 h and 30 min to complete (6.1× relative speedup).

## 4 Conclusion

In this work, we introduced the novel computational problem of computing the gene assignment of an RNA-Seq sample with respect to a set of genes. We also proposed an algorithmic approach to solve this problem and we also implemented it, resulting in the tool, Shark. To the best of our knowledge, Shark is the first tool specifically designed for computing a gene assignment.

We performed an experimental analysis on real data where we evaluated the effectiveness of Shark in speeding up state-of-the-art pipelines for the differential analysis of alternative splicing. Overall, Shark proved to be a preprocessing step that preserves valuable information (i.e. reads) of the selected genes and hence does not negatively affect any downstream pipeline for the differential analysis of alternative splicing. On the other hand, it allows to significantly reduce the size of the input samples, hence it speeds up the pipelines, especially those based on read alignment and read assembly. Furthermore, the efficiency of Shark, combined with an appropriate selection of the different parameters that may be used to influence the performance of the downstream analyses (e.g. the sparsity of the index of STAR), allows to bring all those analyses — nowadays performed on servers — to modern desktop computers.

The accuracy and the efficiency of Shark depend on its parameters *k*, τ and *q* that are the *k*-mer size, the minimum confidence and the base quality threshold, respectively. For this reason, future steps will focus on allowing Shark to automatically estimate the best values of these parameters by exploiting extra information on the read length and the error rate. Furthermore, including also the annotated transcripts in the index or perform a preliminary filtering step using transcript sequences could improve the running times, especially on large gene panels, without affecting accuracy.

Since Shark can be used as a preliminary step in pipelines for the detection of novel alternative splicing events from samples of RNA-Seq data, future work will be devoted to an in-depth experimental analysis of Shark as a preliminary step of a pipeline that includes computationally demanding tools, such as ASGAL (Denti *et al.*, 2018), that relies on mapping reads against a splicing graph, and Trinity (Haas *et al.*, 2013), that assembles RNA-Seq reads, performs transcript abundance estimation and identifies differentially expressed transcripts across samples.

## Supplementary Material

btaa779_Supplementary_Data
