# Supplementary material for: Shark: fishing relevant reads in an RNA-Seq sample
Source: Bioinformatics. 2020 Sep 14;37(4):464–72. doi: 10.1093/bioinformatics/btaa779 (PMC8088329; doi:10.1093/bioinformatics/btaa779)
Supplement: btaa779_Supplementary_Data [file btaa779_supplementary_data.pdf]

Supplemental material for
Shark: fishing relevant reads in an RNA-Seq sample

Luca Denti Yuri Pirola Marco Previtali Tamara Ceccato
Gianluca Della Vedova Raffaella Rizzi and Paola Bonizzoni

1 Exploratory analysis on simulated data

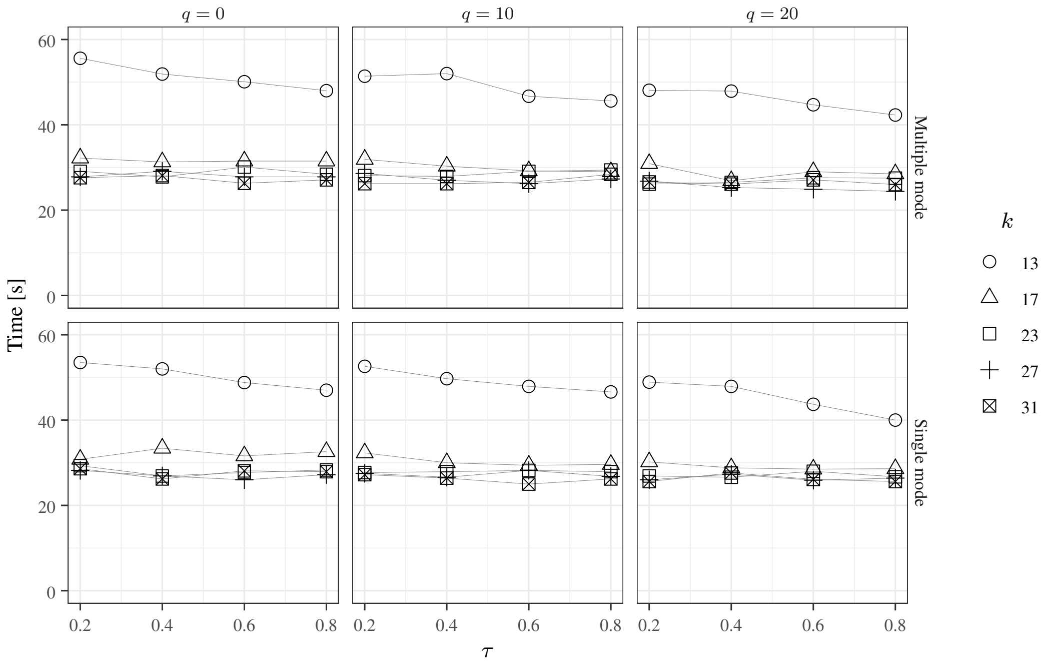

Figure S1: Running times – Exploratory analysis. Times are the average results obtained across the 10 performed runs (seconds). See Table S1 for the complete set of results (including accuracy).

2 Influence of read length and gene size

In a second stage of our experiments on simulated data, we investigated how `Shark` copes with different read lengths and gene sizes to assess whether there are biases induced by these two properties of the input data.

We simulated from the 9403 genes used in Section 3.1 five paired-end RNA-Seq samples of 15 million reads using `Flux Simulator` (Griebel *et al.*, 2012). We considered 5 read lengths between 50bp and 250bp increasing such value by 50bp at each step. We partitioned the set of genes in its quartiles based on their lengths. We obtained four sets of ~ 2276 genes each: the set of small genes, *i.e.,* genes shorter than 707bp (first quartile, Q_1), the set of small-medium genes with length in the interval $(707, 4692]$ (second quartile, Q_2), the set of medium-long genes with length in $(4692, 23863]$ (third quartile, Q_3), and the set of long genes, *i.e.,* genes longer than 23863bp (fourth quartile, Q_4)

Table S1: Accuracy and efficiency results - Exploratory analysis. Accuracy is shown in terms of precision and recall whereas efficiency in terms of time (seconds). Precision, Recall, and Time are the average results obtained across the 10 performed runs. The standard deviation is shown in brackets nearby each value.

			**Multiple mode**						**Single mode**					
k	τ	q	Precision (σ)		Recall (σ)		Time (σ)		Precision (σ)		Recall (σ)		Time (σ)	
13	0.2	0	1.47	(0.63)	99.46	(0.21)	56	(8.70)	1.61	(0.74)	99.29	(0.34)	54	(6.50)
		10	1.43	(0.63)	99.72	(0.07)	51	(6.70)	1.61	(0.74)	99.50	(0.26)	53	(7.60)
		20	1.51	(0.66)	99.72	(0.07)	48	(6.00)	1.75	(0.77)	99.48	(0.29)	49	(6.20)
	0.4	0	3.53	(1.64)	99.08	(0.20)	52	(6.70)	3.61	(1.72)	98.91	(0.33)	52	(6.10)
		10	3.42	(1.58)	99.71	(0.08)	52	(6.80)	3.54	(1.66)	99.47	(0.30)	50	(6.80)
		20	3.97	(1.84)	99.66	(0.05)	48	(5.60)	4.18	(1.96)	99.42	(0.32)	48	(4.00)
	0.6	0	11.23	(5.56)	97.83	(0.20)	50	(5.90)	11.62	(5.90)	97.65	(0.33)	49	(6.30)
		10	10.74	(5.22)	99.56	(0.14)	47	(6.20)	11.31	(5.65)	99.35	(0.33)	48	(6.60)
		20	12.50	(5.62)	99.31	(0.14)	45	(5.50)	13.50	(6.28)	99.07	(0.32)	44	(4.80)
	0.8	0	24.93	(7.77)	91.77	(0.31)	48	(5.30)	26.45	(8.42)	91.60	(0.39)	47	(6.30)
		10	24.25	(7.49)	98.92	(0.26)	46	(5.70)	26.46	(8.36)	98.73	(0.39)	47	(6.70)
		20	26.80	(7.41)	95.75	(0.28)	42	(4.50)	30.08	(8.41)	95.54	(0.41)	40	(4.90)
17	0.2	0	13.72	(4.83)	99.68	(0.06)	32	(4.60)	16.82	(5.77)	99.52	(0.29)	31	(3.70)
		10	12.29	(4.37)	99.74	(0.05)	32	(4.20)	15.54	(5.37)	99.54	(0.29)	32	(4.10)
		20	11.87	(4.20)	99.65	(0.05)	31	(3.10)	16.51	(5.58)	99.45	(0.31)	30	(4.70)
	0.4	0	22.62	(6.72)	99.19	(0.08)	31	(1.40)	25.28	(7.46)	99.04	(0.31)	33	(5.10)
		10	21.67	(6.53)	99.67	(0.06)	30	(2.50)	25.09	(7.43)	99.49	(0.30)	30	(3.40)
		20	21.61	(6.42)	99.47	(0.11)	27	(1.70)	26.72	(7.68)	99.28	(0.31)	29	(2.30)
	0.6	0	30.04	(7.52)	97.71	(0.13)	32	(2.10)	32.49	(8.19)	97.54	(0.34)	32	(2.80)
		10	28.80	(7.35)	99.46	(0.12)	29	(2.10)	32.20	(8.17)	99.28	(0.35)	29	(3.00)
		20	29.80	(7.32)	98.37	(0.16)	29	(2.90)	34.77	(8.32)	98.20	(0.34)	28	(2.50)
	0.8	0	40.21	(7.83)	91.09	(0.28)	32	(4.90)	42.92	(8.43)	90.94	(0.42)	33	(5.10)
		10	39.16	(7.73)	97.91	(0.28)	29	(1.30)	43.07	(8.43)	97.74	(0.42)	30	(2.40)
		20	41.84	(7.73)	90.22	(0.40)	28	(3.50)	47.06	(8.45)	90.04	(0.54)	29	(2.90)
23	0.2	0	12.71	(4.33)	99.56	(0.05)	29	(2.70)	16.68	(5.17)	99.42	(0.30)	29	(3.20)
		10	12.76	(4.34)	99.53	(0.06)	28	(3.40)	17.22	(5.30)	99.35	(0.31)	28	(3.10)
		20	12.74	(4.33)	99.16	(0.07)	26	(1.60)	19.34	(5.78)	98.98	(0.32)	27	(3.50)
	0.4	0	28.04	(7.23)	98.93	(0.08)	28	(2.60)	32.58	(8.10)	98.79	(0.33)	27	(2.90)
		10	26.74	(7.07)	99.43	(0.08)	28	(3.00)	32.48	(8.10)	99.28	(0.33)	28	(2.20)
		20	27.29	(6.98)	98.34	(0.12)	26	(2.90)	35.51	(8.30)	98.17	(0.36)	27	(1.80)
	0.6	0	38.25	(7.78)	97.09	(0.15)	30	(4.30)	41.66	(8.35)	96.93	(0.37)	28	(3.50)
		10	37.02	(7.69)	98.81	(0.16)	29	(4.20)	41.54	(8.38)	98.66	(0.36)	28	(3.40)
		20	39.29	(7.71)	94.37	(0.30)	28	(2.50)	45.57	(8.48)	94.17	(0.47)	28	(2.80)
	0.8	0	52.93	(8.07)	88.85	(0.46)	28	(2.40)	55.66	(8.50)	88.74	(0.60)	28	(3.60)
		10	51.80	(8.02)	93.91	(0.47)	29	(2.90)	55.64	(8.53)	93.76	(0.64)	28	(3.90)
		20	54.34	(8.10)	77.84	(0.72)	28	(4.00)	59.15	(8.56)	77.74	(0.85)	27	(3.10)
27	0.2	0	14.68	(4.62)	99.37	(0.05)	28	(4.30)	19.14	(5.56)	99.22	(0.34)	28	(3.00)
		10	14.87	(4.67)	99.29	(0.07)	29	(3.40)	19.88	(5.73)	99.14	(0.30)	28	(3.60)
		20	15.30	(4.76)	98.42	(0.11)	27	(3.70)	22.81	(6.28)	98.24	(0.36)	26	(2.30)
	0.4	0	32.16	(7.40)	98.66	(0.10)	29	(3.90)	37.80	(8.31)	98.52	(0.36)	27	(3.40)
		10	30.25	(7.23)	99.15	(0.09)	27	(1.80)	37.24	(8.26)	98.99	(0.35)	27	(2.30)
		20	31.44	(7.18)	96.81	(0.19)	25	(2.00)	41.01	(8.44)	96.65	(0.42)	27	(3.60)
	0.6	0	44.00	(7.87)	96.29	(0.20)	28	(2.70)	48.15	(8.49)	96.15	(0.41)	26	(2.00)
		10	42.96	(7.82)	97.88	(0.20)	26	(4.20)	48.13	(8.50)	97.71	(0.40)	28	(5.00)
		20	45.60	(7.91)	90.00	(0.49)	25	(1.60)	52.17	(8.57)	89.85	(0.67)	26	(2.10)
	0.8	0	60.32	(8.36)	85.80	(0.81)	28	(3.10)	62.90	(8.72)	85.70	(0.94)	27	(2.20)
		10	59.20	(8.36)	89.68	(0.85)	27	(2.30)	62.73	(8.73)	89.56	(1.00)	27	(3.20)
		20	61.22	(8.43)	69.67	(0.98)	24	(1.90)	65.37	(8.80)	69.59	(1.08)	26	(3.00)
31	0.2	0	16.73	(5.04)	99.06	(0.08)	28	(2.90)	21.73	(6.04)	98.91	(0.34)	29	(3.00)
		10	17.10	(5.13)	98.94	(0.07)	26	(2.10)	22.72	(6.21)	98.77	(0.34)	27	(2.40)
		20	18.46	(5.35)	96.96	(0.15)	27	(2.70)	26.46	(6.81)	96.78	(0.41)	26	(2.10)
	0.4	0	35.63	(7.39)	98.33	(0.13)	28	(3.00)	42.58	(8.40)	98.16	(0.35)	26	(3.90)
		10	33.28	(7.22)	98.70	(0.13)	26	(4.10)	41.51	(8.34)	98.57	(0.36)	26	(4.60)
		20	34.84	(7.29)	94.82	(0.29)	26	(2.10)	45.48	(8.52)	94.66	(0.50)	28	(2.90)
	0.6	0	51.68	(7.83)	95.18	(0.30)	26	(2.30)	55.79	(8.62)	95.05	(0.49)	28	(3.80)
		10	50.50	(7.83)	96.49	(0.28)	26	(1.60)	55.71	(8.66)	96.37	(0.48)	25	(2.40)
		20	53.37	(7.99)	84.60	(0.74)	27	(2.80)	59.46	(8.74)	84.44	(0.89)	26	(3.40)
	0.8	0	66.25	(9.04)	82.61	(1.13)	27	(6.10)	68.51	(9.36)	82.51	(1.24)	28	(4.10)
		10	65.19	(8.93)	85.54	(1.21)	28	(3.70)	68.23	(9.31)	85.45	(1.32)	26	(3.00)
		20	66.42	(8.92)	62.56	(1.22)	26	(2.80)	69.91	(9.29)	62.50	(1.28)	26	(2.40)

From each quartile we selected 10 random subsets of 100 genes and we evaluated the precision and the recall of `Shark` in assigning reads to the considered set of genes. We ran `Shark` with 4 threads in single-mode setting $k = 17$, $\tau = 0.6$, $q = 10$, and the size of the Bloom filter to 1GB.

Table S2: Accuracy results - Influence of gene size and read length. Accuracy is shown in terms of average precision and average recall obtained across the 10 performed runs. The quartiles refer to specific sets of genes of different length: from the shortest genes (first quartile, Q_1) to the longest ones (fourth quartile, Q_4).

Quartile	Read length	Precision	Recall
Q_1	50	21.42	97.67
	100	35.77	99.66
	150	28.13	99.65
	200	28.11	98.76
	250	25.06	99.22
Q_2	50	35.04	99.71
	100	38.05	99.57
	150	30.33	99.78
	200	32.87	99.83
	250	43.18	99.72
Q_3	50	53.10	99.76
	100	40.57	99.88
	150	33.05	99.87
	200	41.14	99.87
	250	39.21	99.79
Q_4	50	46.38	99.44
	100	44.27	99.60
	150	40.58	99.39
	200	43.31	99.34
	250	40.29	99.77

Table S2 reports the results of this analysis. The first observation is that neither the read length nor the gene size affect negatively the overall accuracy of `Shark`. Indeed, these results mirror the results obtained in the previous analysis (Table S1). In all the cases we tested, `Shark` achieves a very high recall, around 99%, and an overall precision higher than 30%.

The second important observation is that `Shark` is more precise when assigning reads to long genes while still being very sensitive. When considering long genes, the number of k-mers that are indexed in our data structure is higher and this allows our approach to better discern whether a read comes from one of the genes of interest or not, thus increasing its overall precision. However, note that precision is only a secondary goal to our aim since we primarily want that no relevant read is discarded, and that the precision achieved on shorter genes is already sufficient to ensure a significant reduction of the dataset. Indeed, even when considering short genes (quartile Q_1) and 50bp-long reads, `Shark` reduced the input sample from $\sim$15 million pairs to less than 25 000 pairs.

Finally, as expected, both read length and gene size influence the overall efficiency of `Shark`. Indeed, `Shark` was slightly slower and required more memory when indexing longer genes and when analyzing longer reads. However, `Shark` never required more than 1 minute and a half and 2GB of RAM to complete any analysis.

```
ERR_FILE        76
REF_FILE_NAME   /path/to/gtf
GEN_DIR         /path/to/genomefold
READ_NUMBER     10000000
READ_LENGTH     100
PAIRED_END      false
POLYA_SCALE     NaN
POLYA_SHAPE     NaN
TMP_DIR         /path/to/tmp/dir
FASTA           yes
```

Figure S2: `Flux Simulator` parameters used in our exploratory analysis on simulated data.

Table S3: Sizes of the RNA-Seq samples considered in our experimental analysis on real data. The table shows the sizes in terms of number of reads and the uncompressed file size of the 6 original samples and the 6 samples produced by `Shark` after filtering them with respect to the 82 genes involved in the 83 RT-PCR validated events.

	**Original**		**Output by `Shark`**	
**Sample**	No. of reads	Size (GB)	No. of reads	Size (GB)
`SRR1513329`	44 001 064	15.13	952 662	0.20
`SRR1513330`	44 348 240	15.25	985 192	0.21
`SRR1513331`	48 079 644	16.54	1 073 336	0.22
`SRR1513332`	47 492 252	16.15	978 492	0.21
`SRR1513333`	51 140 466	17.60	1 102 950	0.23
`SRR1513334`	45 617 544	15.69	972 840	0.21

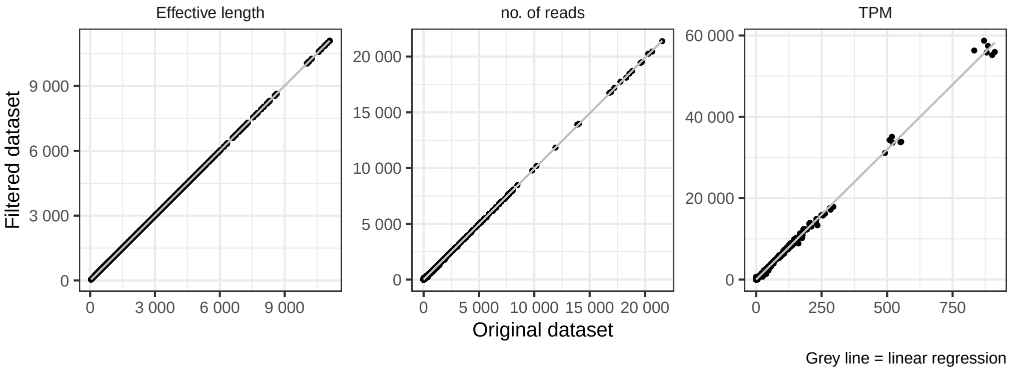

Figure S3: Correlation of the outputs of the transcript quantifier `Salmon` on the full dataset (x-axis) and on the filtered dataset (y-axis) for transcripts annotated in the selected genes.

Table S4: Running times of each step of the `KisSplice` pipeline on the full dataset and on the filtered dataset.

	**Running time** [hh:mm]	
	Full dataset	Filtered dataset
`Shark`		00:38
KisSplice	24:56	02:46
STAR	00:01	00:01
kissplice2refgenome	01:14	00:03
kissDE	00:25	00:00
**Total**	26:37	03:30
